# Honeycomb-Like Hydrogel Microspheres for 3D Bulk Construction of Tumor Models

**DOI:** 10.34133/2022/9809763

**Published:** 2022-02-07

**Authors:** Jiachen He, Chichi Chen, Liang Chen, Ruoyu Cheng, Jie Sun, Xingzhi Liu, Lin Wang, Can Zhu, Sihan Hu, Yuan Xue, Jian Lu, Huiling Yang, Wenguo Cui, Qin Shi

**Affiliations:** ^1^Department of Orthopedics, The First Affiliated Hospital of Soochow University, Orthopedic Institute of Soochow University, 899 Pinghai Road, Suzhou, Jiangsu 215031, China; ^2^National Clinical Research Center for Hematologic Diseases, The First Affiliated Hospital of Soochow University, 899 Pinghai Road, Suzhou, Jiangsu 215031, China; ^3^Department of Orthopaedics, Shanghai Key Laboratory for Prevention and Treatment of Bone and Joint Diseases, Shanghai Institute of Traumatology and Orthopaedics, Ruijin Hospital, Shanghai Jiao Tong University School of Medicine, 197 Ruijin 2nd Road, Shanghai 200025, China; ^4^School of Nanotech and Nano-Bionics, University of Science and Technology of China, 388 Ruoshui Road, Suzhou, Jiangsu 215123, China; ^5^Department of Orthopedics, Wuxi Ninth People's Hospital Affiliated to Soochow University, Wuxi, Jiangsu 214026, China

## Abstract

A two-dimensional (2D) cell culture-based model is widely applied to study tumorigenic mechanisms and drug screening. However, it cannot authentically simulate the three-dimensional (3D) microenvironment of solid tumors and provide reliable and predictable data in response to *in vivo*, thus leading to the research illusions and failure of drug screening. In this study, honeycomb-like gelatin methacryloyl (GelMA) hydrogel microspheres are developed by synchronous photocrosslinking microfluidic technique to construct a 3D model of osteosarcoma. The *in vitro* study shows that osteosarcoma cells (K7M2) cultured in 3D GelMA microspheres have stronger tumorous stemness, proliferation and migration abilities, more osteoclastogenetic ability, and resistance to chemotherapeutic drugs (DOX) than that of cells in 2D cultures. More importantly, the 3D-cultured K7M2 cells show more tumorigenicity in immunologically sound mice, characterized by shorter tumorigenesis time, larger tumor volume, severe bone destruction, and higher mortality. In conclusion, honeycomb-like porous microsphere scaffolds are constructed with uniform structure by microfluidic technology to massively produce tumor cells with original phenotypes. Those microspheres could recapitulate the physiology microenvironment of tumors, maintain cell-cell and cell-extracellular matrix interactions, and thus provide an effective and convenient strategy for tumor pathogenesis and drug screening research.

## 1. Introduction

One of the top 10 challenges in current tumor therapy is to create tumor models that are close to natural tumors, which can interpret the finding efficiently and accurately with cancer therapeutics. Malignant tumors are a severe threat to human health, but far, there are no effective treatment strategies, mainly due to the lack of good tumor research models [1]. At present, research on the tumorigenic mechanism and drug screening mainly rely on the tumor cells cultured on the two-dimensional (2D) plate [2]. However, the microenvironment of solid tumors *in vivo* is three-dimensional (3D), with existing cell-cell and cell-extracellular matrix (ECM) interactions [3, 4]. Therefore, the biological characteristics of tumor cells in the 2D culture system will be changed, which is the main reason for research illusion and drug screening failure [5]. So, it is imperative to develop new tumor models *in vitro* that can simulate the microenvironment of tumor cell growth *in vivo* [6]. The establishment of tumor models *in vitro* reflected the natural microenvironment (*in vivo*) has always been the leading way to tumor research [7]. By simulating the microenvironment of tumorigenesis, we can elucidate the mechanisms related to the progression and metastasis of malignant tumors and find the appropriate treatment from the inherent cause, which is the ultimate goal of establishing tumor models *in vitro* [8]. For this purpose, it is crucial to develop tumor models which more closely resemble the physiological characteristics of tumors under natural conditions [9]. To meet the requirement, several characteristics should be considered: (1) the ability to mimic cell-cell and ECM interactions as *in vivo*; (2) the ability to maintain the inherent characteristics of tumor cells, including stemness, invasiveness, and drug resistance; (3) with a particular spatial structure, which can ensure the exchange of nutrients, cell metabolites, gases, and so on; and (4) batch uniformity that can ensure the repeatability of the results.

In recent years, the drawbacks of two-dimensional culture in tumorigenic mechanism and drug screening research have gradually attracted attention [10]. The horizontal cell culture substrates cannot truly simulate the spatial microenvironment of tumor cells *in vivo* [11]. On the contrary, it will change the inherent characteristics of tumor cells and get unreliable research results [12]. As reported, cells usually exhibit unnatural behavior when inoculated from their resident *in vivo* environment to a horizontal substrate, such as breast epithelial cells that exhibit a tumorigenic tendency when grown on a 3D model that resembles their natural ecological niche but returns to a normal state after transferring to a 2D horizontal substrates [13]. In addition, more and more evidence showed that 3D culture could better simulate the three-dimensional microenvironment in which tumor cells grew *in vivo* and restored the inherent characteristics of tumor cells to a certain extent [14–16]. The tumor models constructed by the 3D culture system mainly include tumor cell spheres and organoids [17]. The former mainly culture tumor cells on ultralow adhesion cell plates, thus forming tumorspheres. However, these kinds of tumorspheres are often highly variable among different batches and cannot be used as a reproducible and reliable way for drugs screening [18, 19]. For the latter, the existing methods on the construction of organoids are challenging to standardize since organic-like material needs to be cultivated in droplets of solidified substrates indispensably. The local differences in physical parameters and accessibility of growth factors result in variations in the shape, size, and distribution of organoids [20]. So, it is challenging to develop a reliable and stable system to decipher the tumor environment. For example, the acidic by-products of PLGA scaffold degradation reduce the pH of the surrounding medium, which is not conducive to cell growth [21, 22]. Therefore, it is of great significance to find suitable materials for a homogeneous 3D culture system conducive to tumor cell adhesion and proliferation, and the biological residues and degradation products of which do not change the biological characteristics of cells [23].

Microfluidic technology promotes the development of material science by precise control of fluid and integration of multiplatforms and provides a powerful tool with integrated, miniaturization, and automation characteristics for high-throughput 3D scaffolds [24, 25]. Hydrogel microspheres prepared by microfluidics have been widely administrated in biomedical research because of their many advantages [26]. One is the homogeneity in structure to ensure convinced results [27]. The second is strong plasticity in component composition that can be made from various raw materials, such as gelatin, hyaluronic acid, and polylactic acid [28]. The third is the diversity of drug loading, of which the drugs can be loaded on the microsphere surface by different methods to achieve the controlled release, such as directly mixing or wrapped by other nanomaterials and subsequently connected to the microspheres by chemical bonding [29–31]. The fourth is large-scale production with low cost and high homogeneity [32]. Apart from that, the microspheres can create a microenvironment suitable for cell growth, nutrient exchange, and even precisely controlled release of drugs [33]. Therefore, hydrogel microspheres prepared by microfluidics have the potential as a 3D growth platform for various types of cells, especially in the construction of tumor models *in vitro* [34]. GelMA is a raw material derived from the extracellular matrix, and the hydrogel microspheres prepared by it can reasonably simulate the microenvironment of tumor cells *in vivo*. Since containing the arginine-glycine-aspartic acid (RGD) motif, it is conducive to the adhesion and proliferation of tumor cells [35]. The lyophilized GelMA microspheres form a porous and honeycomb-like GelMA structure, which can provide a larger space for tumor cell adhesion and growth, nutrient exchange, and cytokine release, as well as suitable mechanical strength. To sum up, GelMA hydrogel microspheres can provide a novel mean for tumor-model construction *in vitro*.

**Scheme 1:**
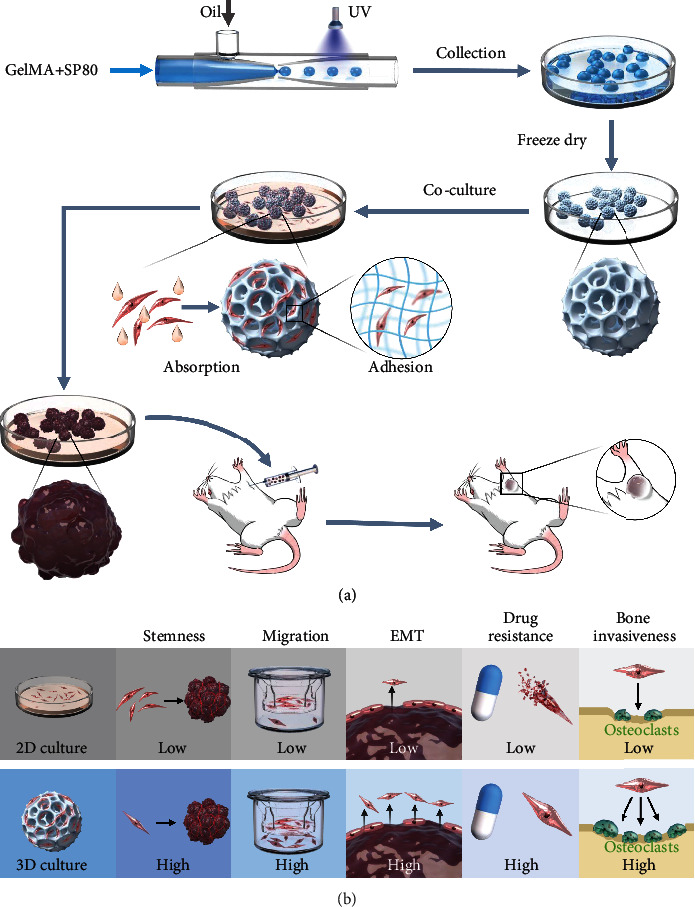
Schematic illustration. (a) Preparation of honeycombed microspheres and its application in tumor model construction. (b) Effects of 3D culture based on honeycombed microspheres on the biological characteristics of tumor cells compared to 2D culture.

Osteosarcoma is a malignant tumor derived from osteoblasts [36]. At present, there is a lack of ideal molecular markers for the early diagnosis and suitable treatment for patients. Such cancer is insensitive to chemotherapy drugs and apt to metastases [37]. The tumor heterogeneity [38] and increased resistance to currently used chemotherapeutic agents in patients [39] give rise to significant difficulties in treating osteosarcoma [40]. In order to explore new and effective drugs, it is essential to develop tumor models of osteosarcoma that can resemble the physiological properties of the natural tumor [41]. In this study, inspired by the honeycomb structure, we constructed the honeycomb-like GelMA hydrogel microspheres by synchronous photocrosslinking microfluidics as 3D scaffolds for culturing osteosarcoma cells (Scheme 1). Hydrogel microspheres can provide a spatial microenvironment for cell-cell and cell-ECM interactions, which are closer to the growth characteristics of natural tumors. So, they can better exhibit the structure and behavior of tumor cells and maintain biological properties and genetic stability. The homogeneity and honeycomb-like microspheres can guarantee the repeatability of the results, and the simple preparation process ensures that they can be manipulated on a large scale.

In this study, we prepared the honeycomb-like GelMA hydrogel microspheres by microfluidic technology, which was a 3D scaffold with uniform structure and size. *In vitro*, the 3D honeycomb-like GelMA microsphere culture system could accelerate cell cycle and proliferation and maintain the cells' stemness, aggressiveness, drug resistance, and angiogenesis compared with that of 2D culture. *In vivo*, osteosarcoma cells obtained from the 3D culture system have shorter tumorigenic cycles, larger tumor volumes, and greater bone aggressiveness than that of 2D culture. These results indicated that the 3D microspheres could well simulate the microenvironment of solid tumors, restore and maintain the biological characteristics of tumor cells, and provide a powerful tool for tumorigenic mechanism and drug screening research.

## 2. Results and Discussion

### 2.1. Construction of Honeycomb-Like GelMA Microspheres

Inspired by the structure of the honeycomb, we constructed the GelMA microspheres with microfluidic technology. Isopropyl myristate solution and different concentrations of GelMA solution containing 1% 2-hydroxy-4′-(2-hydroxyethyl)-2-methylpropiophenone (photoinitiator, PI) were set as the continuous and dispersed phase, respectively. Monodisperse droplets of the same size were prepared in batch at a constant velocity difference and exposed to ultraviolet (UV) light in a synchronous crosslinking tray for photocrosslinking. The droplets were collected. After being washed and freeze-drying, the GelMA microspheres were obtained.

The micromorphology and structure of the microspheres with the different concentrations of GelMA (5%, 7.5%, and 10%) were observed under scanning electron microscopy (SEM). The average diameters of all the microspheres were about 350 *μ*m. However, the pore sizes of the microspheres with different GelMA concentrations were different. The average pore sizes of the microspheres with 5%, 7.5%, and 10% GelMA concentrations were 25.3 ± 6.5 *μ*m, 20.1 ± 5.2 *μ*m, and 7.9 ± 2.6 *μ*m, respectively (Figure S1A, S1B). To explore the effects of honeycomb-like microspheres on cell survival, we observed the surface roughness of microspheres by atomic mechanical microscopy (AFM) at first. With the increase of GelMA concentration, the roughness of the microsphere surface gradually increased (5% GelMA RA = 2.28 nm, 7.5% GelMA RA = 3.48 nm, and 10% GelMA RA = 10.2 nm, Figure S1C). When the concentration of GelMA was 7.5%, the roughness was closer to the natural environment of cell growth, which would be used in the following study [42]. Subsequently, 2 × 10^5^ K7M2 cells (in 1 mL complete medium) were seeded in 10 mg microspheres in 12-well cell culture plates. After 8 hours, the cells had adhered to the microspheres and were added with fresh medium to 3 mL. The results of cell counting showed that the microspheres with 7.5% GelMA concentration were more favorable for K7M2 cell proliferation (fold change of 5%, 7.5%, and 10% GelMA was 1.95, 2.52, and 1.1, respectively), indicating that the microspheres prepared with 7.5% GelMA were more conducive to the growth of mouse osteosarcoma cells (Figure S1D). Therefore, we prepared the microspheres with 7.5% GelMA for the subsequent study.

### 2.2. Characterization of Honeycomb-Like GelMA Microspheres

**Figure 1:**
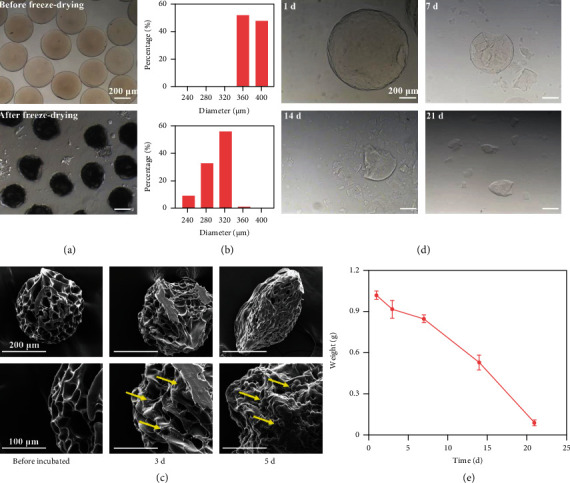
Characterization of the honeycomb-like GelMA microspheres. (a) Gross view of the microspheres before and after freeze-drying. (b) Particle size distribution of the microspheres before and after freeze-drying. (c) The microspheres cultured with osteosarcoma K7M2 cells after dehydration were observed by SEM (yellow arrows represented the cell infiltration). (d) Degradation curve of the microspheres. (e) Quantitative analysis of degradation (*n* = 3).

After freeze-drying, the microspheres were observed by optical microscopy. The images showed that the microspheres were uniform in size and regular in shape before or after freeze-drying. Their diameters were significantly reduced after freeze-drying. The average diameter shrank from 381 ± 12.3 nm to 300 ± 26.7 nm. Acceptably, the diameter of the microspheres returned to the size that nearly before lyophilization after absorbing water, and this swelling feature facilitated the cells to adhere to its interior (Figures 1(a) and 1(b)). The SEM images showed that many pores were connecting the surface and interior of the microspheres, which was conducive to nutrient exchange, cell growth, and migration. After 3 and 5 days of cell inoculation, we found that the cells could not only grow on the surface of microspheres but also migrate into their interior (Figure 1(c)). Therefore, the honeycomb-like structure could provide an ample space for cell adhesion and growth and enhance the cell-cell and cell-ECM interactions, which can better simulate the microenvironment of tumor cell growth *in vivo*.

As a hydrogel material derived from ECM, GelMA has been widely used in medical biomaterials, such as hydrogel microspheres loaded with NK cells for tumor killing and loaded with nucleus pulposus cells for treating disc degeneration, probably due to its good biocompatibility [26]. To evaluate the biocompatibility of GelMA, the cytoskeleton and Live/Dead staining of osteosarcoma MG63 cells cultured on the microspheres were performed on days 1, 3, and 5. The results showed that the cells could grow and stretch well, further indicating that they had good biocompatibility (Figure S2). Subsequently, the microspheres were immersed in PBS containing 2 U/mL type II collagenase in a 37°C shaker; then, the weight and morphology were recorded weekly. The degradation of the microspheres was approximately 80% after 28 days (Figures 1(d) and 1(e)). To sum up, the honeycomb-like microspheres fabricated by microfluidics are porous, degradable, and biocompatible 3D scaffolds, which were suitable for 3D cell culture, especially for the construction of tumor models *in vitro*.

### 2.3. Proliferation of Tumor Cells Cultured in Microspheres

Maintaining the original biological state of cells is crucial for establishing 3D culture models of tumors. The maintenance of tumor cell stemness is not only the basis for studying the mechanism of tumorigenesis but also essential for tumor drug screening. To investigate the effect of the 3D microsphere cell culture on the biological characteristics of osteosarcoma cells, we incubated K7M2 cells in 3D microspheres (MS) or 2D tissue culture plates (TCP), respectively. After seven days, the cells were harvested. Some of the cells were used to detect the cell cycle by flow cytometry, and the others were used to extract RNA and protein for detection of the related gene and protein expression.

**Figure 2:**
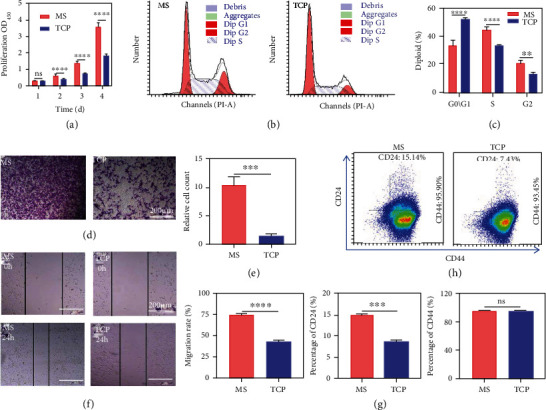
3D microsphere cell culture promoted osteosarcoma cell proliferation and enhanced the osteosarcoma cell migration. K7M2 cells were cultured on MS and TCP for 7 days, respectively. (a) Proliferation of K7M2 cells by the CCK-8 kit. (b, c) Cell cycle and quantitative results of K7M2 cells by FCA. (d, e) The Transwell assay and quantitative results of the migratory ability of K7M2 cells. (f, g) Scratch assay and quantitative results of migratory ability of K7M2 cells. (h) Protein expression and quantitative results of CD24 and CD44 in K7M2 cells (*n* = 3; ^∗∗^*p* < 0.01, ^∗∗∗^*p* < 0.001, and ^∗∗∗∗^*p* < 0.0001; ns: not significant).

For the cell proliferation, the collected K7M2 cells were further cultured in the 96-well plate with the same cell number, and the cell viability was detected by the cell counting kit-8 (CCK-8 kit). As expected, the proliferation rate of K7M2 cells in MS culture was significantly higher than that in the TCP group at each time point (Figure 2(a)). The maintenance of stemness is closely related to tumor cell proliferation, self-renewal, and tumor formation. Sox2 and Nanog are core factors in the transcriptional regulation of embryonic stem cells (ESCs). Sox2 is a high swimming class nonhistone cassette structural domain protein, which stabilizes pluripotency of ESCs by maintaining appropriate expression levels of Oct4. Nanog is a homologous cassette structural domain protein that can block the differentiation of ESCs. Nucleostemin plays an essential role in maintaining cell proliferation, cell cycle regulation, telomere stability, genomic integrity, and self-renewal of stem cells and tumor cells. Thus, we examined these genes of K7M2 in two different cultural ways. The mRNA expression of Sox2, Nanog, and Nucleostemin in the MS group was increased 79.2, 4.68, and 6.17 times than that of the TCP group, respectively (Figure S3), suggesting that MS culture increased the tumor cell stemness. We further investigated the cell cycles of K7M2. Compared to 2D culture, K7M2 in MS culture showed a decreased percentage in G0/G1 phase and an increased percentage in S and G2/M phases. These results demonstrated that MS culture promoted DNA synthesis and induced cell cycle shift from G1 to S phase in K7M2 cells, which further revealed an enhanced proliferation capacity of the cells from another view (Figures 2(b) and 2(c)).

### 2.4. Migration of Tumor Cells Cultured in Microspheres

Osteosarcoma metastasis is a significant cause of osteosarcoma-related death. Many studies have shown that the five-year survival rate among osteosarcoma patients with secondary lung metastases is 23%, and the four-year survival rate with secondary bone metastases is close to zero [37]. To investigate the migration ability of osteosarcoma cells, K7M2 cells derived from MS and TCP culture conditions were seeded into the upper chamber of the Transwell system for 24 hours. The migrated cells were stained by crystalline violet and observed under a light microscope. The results showed that 3D-MS culture promoted the migration of K7M2 cells (Figures 2(d) and 2(e)). The scratch assay was performed to simulate the rate of “wound healing” in response to the migration ability of the cells, and the results also displayed that the “healing” ability of K7M2 cells was enhanced by MS culture (Figures 2(f) and 2(g)).

During the formation and changes of the tumor microenvironment, normal fibroblasts are transformed into cancer-associated fibroblast (CAF), an essential component of the tumor-stromal microenvironment, under the stimulation of many chemokines. Fibroblast-activated protein alpha (FAP*α*) is a specific surface marker of CAF, which can enhance the tumor cell invasion along with the fiber by promoting stromal reconstruction and participating in signal transduction, such as vascular endothelial growth factor (VEGF). It also participates in tumor angiogenesis to form a tumor biological barrier and inhibit the function of effector T cells, thus promoting tumor progression. VEGF is a specific growth factor that promotes vascular endothelial cell migration and facilitates extracellular matrix degeneration, acting similarly to matrix metalloproteinase-2 (MMP-2). CXCR3 is a chemokine receptor that promotes tumor development by accelerating the rate of angiogenesis within local tissues. We verified the enhanced migration ability of K7M2 cells seeded in MS culture was relevant to the increased genetic level of migration-related genes, FAP*α*, VEGF, MMP-2, and CXCR3 (FAP*α* increased 1.69 times, VEGF increased 6.56 times, MMP-2 increased 1.42 times, and CXCR3 increased 2.28 times, respectively, Figure S4).

Since K7M2 cells from the MS group possessed a higher migration ability than those from the TCP group, we further analyzed the expression of CD24 and CD44 of K7M2 cells by flow cytometry assay. The results showed that CD24 expression was significantly elevated in the MS group, but there was no significant difference in the expression of CD44 (Figure 2(h)). CD24 is a salivary glycoprotein expressed in most B lymphocytes and neuronal cells but also in neutrophils and neutrophil precursors. CD44 is a transmembrane glycoprotein that is mainly found on the cell surface, and its central role is to dare the adhesion of tumor cells. Both of them are expressed on some tumor cells and associated with tumor invasion and metastasis. Thus, the elevated expression may be due to MS that simulates the tumor microenvironment to maintain the malignant behaviors of osteosarcoma cells.

### 2.5. 3D Culture Promoted EMT and Drug Resistance of Osteosarcoma Cells

**Figure 3:**
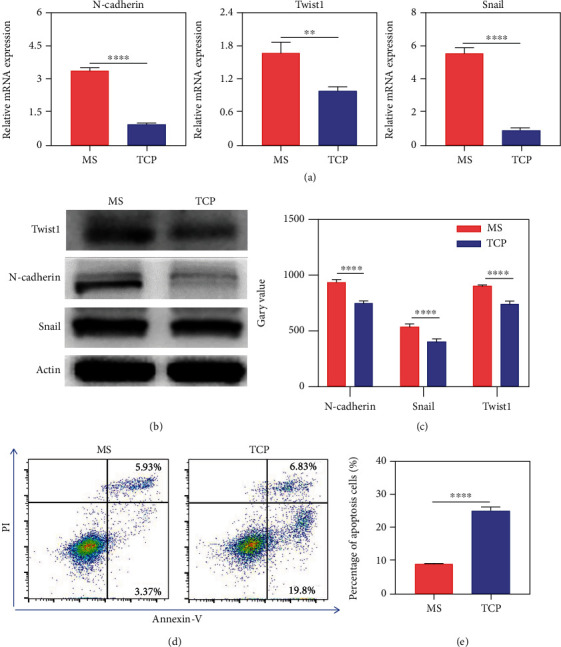
3D microsphere cell culture improved EMT-related molecule expression and drug resistance in osteosarcoma cells. K7M2 cells were cultured on MS and TCP for 7 days, respectively. (a) Gene expression of N-cadherin, Twist1, and Snail in K7M2 cells by qRT-PCR. (b, c) Protein expression and quantitative results in K7M2 cells by western blot. (d, e) apoptosis and quantitative results of K7M2 cells treated with Dox for 24 hours by FCA (*n* = 3; ^∗∗^*p* < 0.01, ^∗∗∗^*p* < 0.001, and ^∗∗∗∗^*p* < 0.0001).

Epithelial-mesenchymal transformation (EMT) is a complex biological process, which is associated with tumor progression, invasion, and metastasis. Many studies had demonstrated that N-cadherin, Snail, and Twist1 were closely related to EMT. Therefore, the mRNA and protein expression levels of N-cadherin, Snail, and Twist in 2D and 3D cultured K7M2 cells were detected, respectively. Impressively, the mRNA and protein expression of these three molecules were significantly increased in MS cultured cells. The gene and protein levels of N-cadherin were increased by 3.39 and 1.26 times, Snail by 5.6 and 1.3 times, and Twist1 by 1.69 and 1.2 times, respectively (Figures 3(a)–3(c)). It is an art-of-state that the MS system may be favorable to the EMT process and associated with tumor anisotropy. Through EMT, epithelial cells lose their epithelial phenotype such as cell polarity and connection to the basement membrane and gain a higher mesenchymal phenotype such as migration and invasion, to degrade the extracellular matrix and resistance to apoptosis.

To further investigate the influence of the MS culture system on the biological characteristics of K7M2 cells, AnnexinV staining was performed to detect the sensitivity of cells derived from two culture methods to doxorubicin (DOX, 50 nmol/mL) which is a first-line chemotherapeutic drug for the treatment of osteosarcoma. Since AnnexinV is a specific marker of early cell apoptosis, the results showed that the early apoptotic cells cultured in 3D-MS was only 3.14%, compared to 21.6% in that of 2D-TCP, suggesting that 3D-MS culture endowed the tumor cells with resistance to chemotherapeutic drugs (Figures 3(d) and 3(e)). Therefore, the 2D culture of tumor cells may lead to the false-positives in drug screening, while 3D-MS culture is conducive to improve the effectiveness of drug screening, which is of great significance for the precise tumor treatment and new drug discovery.

To further verify the potential of honeycomb-like microspheres in culturing tumor cells for drug screening, we conducted the same exploration on the human osteosarcoma cell line (MG63). After being cultured in 3D-MS for seven days, MG63 cells were further treated with 50 nmol/mL DOX for 24 hours. The early apoptosis of the cells cultured in MS culture was only 23.9%, while that of the TCP group was 43.1% (Figure S5A). The antiapoptotic ability was significantly improved. Cell apoptosis is regulated by many apoptosis-related molecules. The balance between antiapoptotic molecules and proapoptotic molecules determines the final fate of the cells. B-cell lymphoma-2 (BCL-2) and BCL-2-associated x protein (Bax) are the prominent members that regulate cell apoptosis. BCL-2 enhances cell resistance to DNA damage factors and apoptosis caused by chemotherapeutic drugs. When Bax is overexpressed, it inhibits the antiapoptotic effect of Bcl-2 through the formation of homodimer Bax/Bax, thereby promoting cell apoptosis. The mRNA expression of BCL-2 in the 3D-MS cultured MG63 cells was 3.22 times higher than that of 2D-TCP, while the expression of Bax had not changed (Figure S5B). These results further verified that 3D-MS culture could enhance the antiapoptotic ability of tumor cells, which was an essential parameter for evaluating drug screening in practice.

### 2.6. 3D-MS Culture Enhanced Osteoclastogenesis of Tumor Cells

Many studies have shown that cancer cells have a solid ability to induce osteoclastogenesis, and osteosarcoma is often accompanied by osteopenia and osteoporosis. Therefore, we prepared the conditioned medium with the supernatants of K7M2 cells cultured in 3D-MS and 2D-TCP and further investigated its effect on osteoclast formation, respectively.

**Figure 4:**
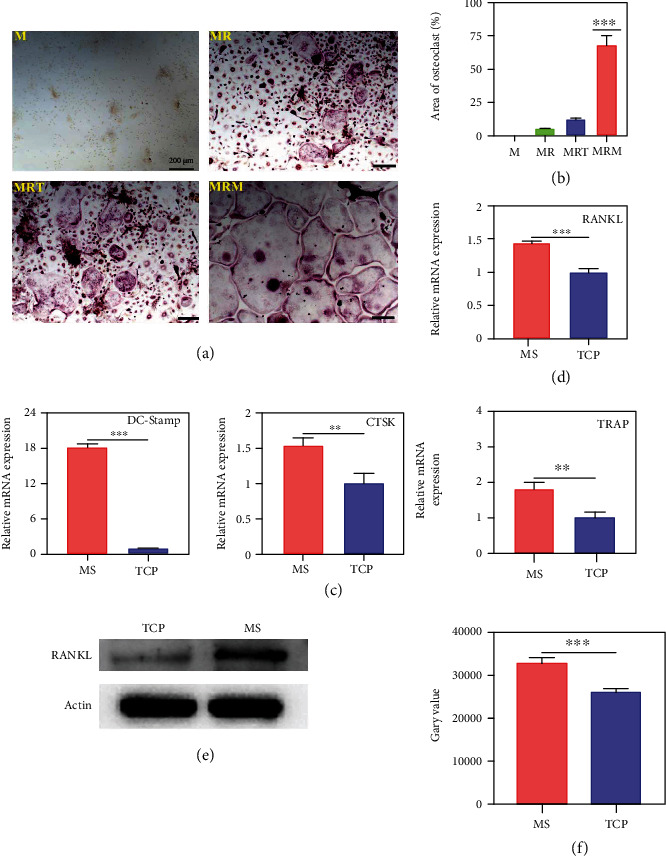
3D microsphere cell culture boosted osteosarcoma cells to activate BMM-derived osteoclastogenesis. K7M2 cells were cultured on MS and TCP for 7 days, respectively. BMMs were induced into osteoclasts in addition with K7M2 cell supernatant for 5 days. (a, b) TRAP staining and quantitative results of mouse BMM-derived osteoclasts (M: M-CSF; MR: M-CSF+RANKL; MRT: M-CSF+RANKL+TCP supernatant; M-CSF+RANKL+MS supernatant). (c) Gene expression of DC-stamp, CTSK, and TRAP in mouse BMM-derived osteoclasts by qRT-PCR. (d, e) Gene and protein expression of RANKL in K7M2 cells by qRT-PCR and WB. (f) Quantitative analysis of RANKL protein (*n* = 3; ^∗∗^*p* < 0.01 and ^∗∗∗^*p* < 0.001).

The TRAP staining displayed that the supernatant from the 3D-MS culture promoted more multinuclear osteoclast formation than that of 2D-TCP culture (Figures 4(a) and 4(b)). In addition, qPCR results also showed that the supernatant of 3D culture promoted the expression of osteoclast-related genes, including DC-STAMP, CTSK, and TRAP (Figure 4(c)). Therefore, we hypothesized that osteosarcoma cells secreted osteoclastogenic factors, while 3D-MS culture enhanced the secretion of these factors. RANKL is a critical factor in the differentiation of osteoblasts, promoting the transcription and expression of osteoblast-related genes. Compared to the K7M2 cells cultured on 2D plates, the cells in MS culture expressed significantly higher RANKL gene and protein (Figures 4(d)–4(f)). It is evident that MS promotes osteosarcoma cells to secrete more factors and contribute to the bone erosion induced by osteosarcoma.

Finally, we performed RNA sequencing (RNA-seq) to examine transcriptome profiles in the cells of 3D-MS and 2D-TCP culture. The results further validated the upregulated expression of osteoclast-related genes and cancer-related genes (Figure S6).

### 2.7. Osteosarcoma Cells Cultured in 3D-MS Have Stronger Tumorigenicity *In Vivo*

**Figure 5:**
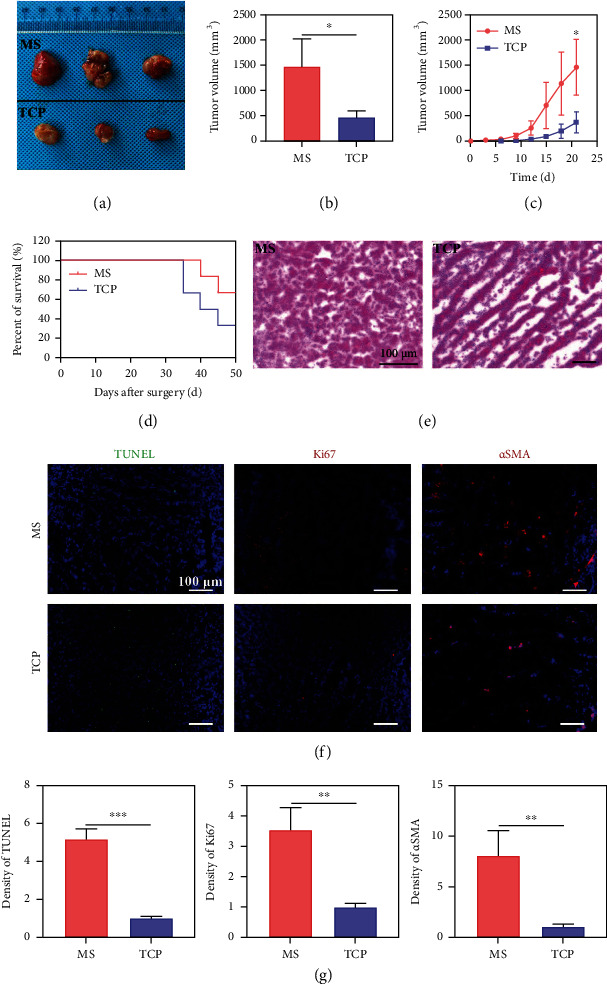
3D microsphere cell culture accelerated osteosarcoma formation in mice. K7M2 cells were cultured on MS and TCP for 7 days and collected, respectively. Mice were implanted with K7M2 cells from MS or TCP culture, respectively. (a, b) Gross view and quantitative results of tumors. (c) Tumor growth curves. (d) Survival curves of mice. (e) H&E staining of tumor tissues. (f, g) TUNEL staining, Ki67 and *α*SMA expression, and quantitative results of tumor tissues by IHC (*n* = 6; ^∗^*p* < 0.05, ^∗∗^*p* < 0.01, and ^∗∗∗^*p* < 0.001).

*In vitro* 3D-MS cultured osteosarcoma cells preferably maintain their original properties, including tumor stemness, migration, drug resistance, and bone erosion. Next, we further clarified the characteristics of osteosarcoma cells upon two culture conditions *in vivo*. K7M2 cells (1 × 10^6^ cells/mouse) were implanted subcutaneously into 6-week-old female BALB/c mice, and the tumor volumes were recorded every three days. Tumor bulges could be observed with naked eyes on the 7th day after mice inoculated with 3D-cultured tumor cells, while it takes up to 13 days for 2D-cultured cells. Tumor formation and growth rate of the MS group were faster than those of the TCP group under the same time point (Figures 5(a)–5(c)), and the survival curve showed that the mortality of mice in the 3D-MS group was higher (Figure 5(d)) than that of the 2D-TCP group. After 28 days, partial of the experimental mice in each group were taken for sampling and analysis. The tumor size implanted with 3D-MS cultured cells was significantly more significant than that of 2D-TCP culture. Morphologically, the tumor tissues of the MS group were more compact, while the tumor tissues of the TCP group had more patchy necrotic areas (Figure 5(e)). Tunnel and immunohistochemical staining (IHC) for Ki67 and smooth muscle actin*α* (*α*SMA) showed that the specimens of the MS group had less apoptosis and more cell proliferation, compared with that of the TCP group (Figures 5(f) and 5(g)), indicating osteosarcoma cells cultured in MS had stronger tumorigenicity.

### 2.8. Osteosarcoma Cells Cultured in 3D-MS Promoted Bone Loss *In Vivo*

Due to the ability of osteosarcoma cells to induce osteoclastogenesis, osteosarcoma patients often have the complication of osteopenia, which increases mortality. So, we analyzed the bone mass of the distal femur by micro-CT scanning in the tumor-bearing mice. Micro-CT scanning showed that the bone mass density (BMD) of the mice implanted with K7M2 cells from 3D-MS was much lower than that of 2D-TCP (Figure S7A, S7B). Meanwhile, the mice showed a significant decrease in bone volume/tissue volume (BV/TV) and trabecular thickness (Tb. Th), and a significant increase in the ratio of bone surface area to bone volume (BS/BV) parameters. All the bone mass parameters indicated that 3D-MS cultured K7M2 cells were more capable of bone erosion, which may be owing to the cells secreted more RANKL.

In summary, 3D-MS cultured K7M2 cells displayed stronger oncogenicity than that from 2D-TCP culture, mainly manifested as shorter tumor formation time, larger tumor size, higher proliferation rate and mortality, and more substantial bone erosion tumor-bearing mice. Therefore, we believe that the *in vitro* tumor model constructed based on 3D honeycomb-like GelMA hydrogel microspheres can restore the original characteristics of the primary tumors. With the RGD motif of GelMA, cells could attach to the hydrogel microspheres fast and stably. Meanwhile, the 3D structure of microspheres establishes an ECM environment similar to the solid tumors *in vivo*. It allows us to acquire more accurate results than 2D cell culture for drug screening and predicting the response to tumor treatment, thereby improving tumor precision medicine.

## 3. Conclusion

In this study, we constructed the honeycomb-like porous GelMA hydrogel microspheres by microfluidic technology and applied them to culture osteosarcoma cells. *In vitro* and *in vivo* studies showed that 3D structural microspheres could preferably maintain the biological properties and tumorigenicity of osteosarcoma cells, since honeycomb-like porous GelMA hydrogel microspheres provide an environment close to the actual tumors. Thereby, it provides a novel viable option for precision medicine and drug screening to explore new therapeutic means for osteosarcoma.

## 4. Materials and Methods

### 4.1. Materials and Cells

Gelatin was purchased from Zhejiang Luosailuo Co. (China). Methacrylate anhydride was purchased from Aladdin Reagent Co. (China). All flow cytometry antibodies were purchased from Peprotech (USA). Dulbecco's modified Eagle's medium (DMEM), fetal bovine serum (FBS), trypsin, and penicillin/streptomycin were purchased from Gibco (USA). K7M2 cell line and MG63 Cell line were purchased from the Chinese Academy of Sciences (Shanghai, China). All reagents were of analytical reagent grade and used as received.

### 4.2. Animal Ethics

All-female 6-week-old BALB/c mice in the study were purchased from Beijing SPF Biotechnology Co., Ltd. (Production License No: SCXK (Beijing) 2019-0010). The animal research protocol was reviewed and proved by the Experimental Animal Ethics Committee of Soochow University.

### 4.3. Preparation of GelMA

GelMA was synthesized according to a previous protocol [43]. Firstly, 40.0 g gelatin was dissolved in 400 mL PBS (50°C), and the dissolution was accelerated by a magnetic agitator. Then, 32.0 mL methacrylate anhydride was added and diluted five times with PBS to stop the reaction. At last, the solution was dialyzed with a dialysis membrane for two weeks (molecular weight cut off 10 kDa), and ddH_2_O was replaced daily to fully remove the free methacrylic anhydride until the liquid was clarified.

### 4.4. Preparation of Microspheres

5%, 7.5%, or 10% *w*/*v* GelMA solution mixed with 1% PI was used as the dispersed phase and isopropyl myristate as the continuous phase, and the two phases were injected at different speeds by a microinjection propulsion pump. The dispersed phase formed a single continuous liquid under the action of shear force, with different droplet sizes formed at different speed differences. The collected droplets were crosslinked by 6.9 mWcm^−2^ UV for 1 min, washed by ethanol and ddH_2_O, and then freeze-dried to form porous GelMA hydrogel microspheres.

### 4.5. Characterization of Microspheres

The microspheres were fixed to SEM sample stubs using conductive tape and then gold sputter-coated for 45 s (Quorum Technologies, SC7620, UK). The morphology was observed by SEM, at an acceleration voltage of 10 kV (SEM, Hitachi, S-4800, Japan). The mean diameter and pore size of 100 randomly selected microspheres were measured using Nano Measure1.2 software (Shanghai, China). The surface roughness of the honeycomb microspheres was observed by Atomic Force Microscopy (AFM Bruker, Germany).

### 4.6. Biodegradability and Biocompatibility of Microspheres

1 g of microspheres was added into 10 mL PBS solution (with 2 U/mL collagenase II) and placed in a constant temperature shaker at 37°C. The microspheres were weighed (Wt) after 1, 2, and 3 weeks after drying, and the residual weight (%) was calculated using the formula Wt/W0. The experiment was repeated three times simultaneously. The microspheres were cocultured with K7M2 cells and treated with alcohol gradient dehydration on the third and fifth days. The microspheres were observed under SEM. In addition, microspheres were cocultured with MG63 cells. Skeletal and dead staining was performed at 1, 3, and 5 days, respectively, and observed by inverted fluorescence microscopy and confocal laser microscopy (Leica, German).

### 4.7. Cell Proliferation Assay

K7M2 cells were cultured in microspheres (MS) and tissue cell plates (TCP) for seven days. Cells were harvested and pooled in 96-well plates at a concentration of 1 × 10^3^ cells per well. At the indicated time points (1, 2, 3, and 4 days), CCK-8 reagent (Dojindo, Japan) was added in the culture medium at a ratio of 10% (*V*/*V*). After 2-hour incubation, 100 *μ*L of medium was pipetted into a fresh 96-well plate, and the absorbance at 450 nm was measured using a microplate reader (BioTek, USA).

### 4.8. Gene Expression

K7M2 cells were incubated in MS and TCP for seven days and collected. The gene expression was analyzed by semiquantitative RT-PCR. Glyceraldehyde-3-phosphate dehydrogenase (GAPDH) was applied as an endogenous reference gene. The 2^-*ΔΔ*t^ method was used for quantization. Primer sequences were designed by Genewiz and are summarized in Table 1.

### 4.9. Cell Cycle Assay

K7M2 cells were cultured for seven days, then harvested and detected by flow cytometer assay (FCA) with cell cycle detection kits (Kaiji Biology, China). The results were analyzed by Moditfit 5.0 (Verity Software House, USA).

### 4.10. Cell Apoptosis

After K7M2 cells were cultured for seven days and collected, the cells were pooled in 12-well plates at a concentration of 3 × 10^5^ per well. Cells were treated with DOX (50 nmol/mL) for 24 hours. Then, cell apoptosis was assayed by FCA with the apoptotic kit (BD Biosciences, USA).

### 4.11. Western Blot

The harvested cells were lysed, and the supernatant was collected. The protein samples were separated on a 10% SDS gel and transferred to a nitrocellulose membrane. The primary antibodies were incubated with the membrane overnight at 4°C. Then, membranes were incubated with corresponding secondary horseradish peroxidase-conjugated antibodies for 2 hours. The target proteins were detected employing strengthened chemiluminescence reagents (Thermo Fisher Scientific, USA) and then imaged with the ChemiDoc™ Touch Imaging System (Bio-Rad Laboratories, USA). Quantification of the band intensity was performed using ImageJ software (NIH, USA).

### 4.12. FCA Assay

After seven-day culture, K7M2 cells were stained with anti-CD24 and CD44 antibodies (BioLegend, USA) for FCA analysis. All flow cytometry was performed on a Beckman flow instrument and analyzed using FlowJo 10.

### 4.13. Tartrate Phosphatase (TRAP) Staining

We isolated BMMs (Bone Marrow-derived Macrophages) from mouse femur and induced them with M-CSF (30 ng/mL, R&D, USA) and RANKL (50 ng/mL, R&D, USA) to form osteoclasts as described previously [44]. For MRT and MRM groups, TCP and MS group supernatants were added at a ratio of 1 : 10 (DMEM : supernatant) at each fluid change, respectively. Osteoclasts in the 48-well plate were fixed with 4% paraformaldehyde for 30 minutes and then washed with PBS for three times. TRAP dye was added and placed in a 37°C thermostatic oven for incubation for 30 minutes and observed under a microscope.

### 4.14. RNA-seq

The transcriptome sequencing was conducted by OE Biotech Co., Ltd. (Shanghai, China). Total RNA was extracted using the TRIzol reagent according to the manufacturer's protocol. RNA purity and quantification were evaluated using a NanoDrop 2000 spectrophotometer (Thermo Scientific, USA). RNA integrity was assessed using the Agilent 2100 Bioanalyzer (Agilent Technologies, Santa Clara, CA, USA). Then, the libraries were constructed using the TruSeq Stranded mRNA LT Sample Prep Kit (Illumina, San Diego, CA, USA) according to the manufacturer's instructions. The libraries were sequenced on an Illumina HiSeq X Ten platform, and 150 bp paired-end reads were generated. Raw data (raw reads) of fastq format were firstly processed using trimmomatic; about 56.67 M raw reads for each sample were generated. About 54.56 M clean reads for each sample remained, which were obtained for downstream analyses by removing reads containing adapter, reads containing ploy-N, and low-quality reads from raw data. The clean reads were mapped to the human genome using HISAT2.

### 4.15. *In Vivo* Study

After wiping the right axilla of the mice with 75% alcohol disinfectant, 5-week-old BALB/c female mice were injected subcutaneously with 1 mL syringe with 1 × 10^6^ cells/mL of K7M2 cell suspension under two culture conditions in 100 *μ*L PBS, and the tumor growth of the mice was observed every three days. The observation was performed for 21 days after the growth of the tumor. The tumor size was estimated by measuring its length and width. After 21 days, animals are sacrificed, and tissues are collected for histopathological examination.

### 4.16. Histopathology

Tumor tissues were collected and fixed in buffered 4 wt% formalin, followed by dehydration and embedded in paraffin. Serial sections of 5 *μ*m in thickness were acquired by a microtome and were prepared for hematoxylin-eosin (H&E), TUNEL, Ki67, and *α*SMA (Abcam, UK) immunofluorescent staining.

### 4.17. Microcomputed Tomography (*μ*CT) Scanning

The femurs harvested from two groups were scanned using the *μ*CT system (SkyScan 1176, Belgium), as previously described. The samples were scanned at a high resolution (9 *μ*m) with an energy of 50 kV, 200 mA of intensity, and regular increments of 0.7 in the 180° rotational steps. Three-dimensional (3D) reconstruction was performed using NRecon, and images were visualized using Mimics v10.01 software (Materialise, Belgium). The bone parameters, such as the percentage of bone volume out of total tissue volume (BV/TV, %), bone mineral density (BMD, mg/cm^3^), ratio of bone surface area to bone volume (BS/BV, %), and trabecular thickness (Tb. Th, mm), were analyzed by CT Analyzer (CTAn, SkyScan, Belgium).

### 4.18. Statistical Analysis

All data are recorded as the mean ± standard deviation. Statistical analysis was performed with an unpaired two-tailed Student's *t*-test for single comparisons with GraphPad Prism 8 (GraphPad Software, USA). One-way analysis of variance (ANOVA) was used to compare data from more than two groups. Differences were considered significant at *p* < 0.05.

## Figures and Tables

**Table 1:** Sequences of mouse primer for qRT-PCR.

Gene	Primer forward (5′-3′)	Primer reverse (3′-5′)
Nanog	CACAGTTTGCCTAGTTCTGAGG	GCAAGAATAGTTCTCGGGATGAA
Sox-2	GCGGAGTGGAAACTTTTGTCC	GGGAAGCGTGTACTTATCCTTCT
Nucleostemin	AAAGCGAGTAAACGTATGACCTG	AGCACTATTTGGAACACCTGG
VEGF	GCACATAGAGAGAATGAGCTTCC	CTCCGCTCTGAACAAGGCT
FAP*α*	CCAGTTCCAGAAATGATAGCC	GACAGGACTGAGACATTCTGC
CXCR-3	GGTTAGTGAACGTCAAGTGCT	CCCCATAATCGTAGGGAGAGGT
MMP-2	TGGCAAGTACGGCTTCTGTC	TTCTTGTCGCGGTCGTAGTC
N-cadherin	TCAGGCGTCTGTAGAGGCTT	ATGCACATCCTTCGATAAGACTG
Twist1	GGACAAGCTGAGCAAGATTCA	CGGAGAAGGCGTAGCTGAG
Snail	CACACGCTGCCTTGTGTCT	GGTCAGCAAAAGCACGGTT
DC-STAMP	TCCTCCATGAACAAACAGTTCCAA	AGACGTGGTTTAGGAATGCAGCTC
CTSK	AATACCTCCCTCTCGATCCTACA	TGGTTCTTGACTGGAGTAACGTA
TRAP	AGACCCAATGCCACCC	GGACCTCCAAGTTCTTATC
RANKL	CGCTCTGTTCCTGTACTTTCG	GAGTCCTGCAAATCTGCGTT
Bcl-2	GGATAACGGAGGCTGGGAT	GGCAGGCATGTTGACTTCAC
Bax	GAGGATGATTGCCGCCGTGGACA	GTCCACGGCGGCAATCATC
GAPDH	CTGAACGGGAAGCTCACTGG	TGAGGTCCACCACCCTGTTG

## Data Availability

All data used to support the findings of this study are available from the corresponding author upon request.

## Supplementary Materials

Supplementary Materials Figure S1: the morphological characteristics of the hydrogel microspheres with different GelMA concentrations at 5%, 7.5%, and 10%. (A) SEM observation of the microspheres. (B) Particle size distribution of the microspheres. (C) AFM detection of roughness of the microspheres. (D) Cell counting of osteosarcoma cells cultured in the microspheres for 3 days (*n* = 3; ^∗^*p* < 0.1, ^∗∗^*p* < 0.01, and ^∗∗∗^*p* < 0.001). Figure S2: fluorescent images for human osteosarcoma MG63 cells cultured in the GelMA microsphere for various periods. (A) The nucleus was stained blue, and the cytoskeleton was stained green (magnification, 50x). (B) Dead cells were stained red, and live cells were stained green (magnification, 50x). Figure S2: fluorescent images for human osteosarcoma MG63 cells cultured in the GelMA microsphere for various periods. (A) The nucleus was stained blue, and the cytoskeleton was stained green (magnification, 50x). (B) Dead cells were stained red, and live cells were stained green (magnification, 50x). Figure S4: K7M2 cells were cultured on MS and TCP for 7 days, respectively. Gene expression of VEGF, Fap*α*, CXCR3, and MMP-2 in K7M2 cells by qRT-PCR (*n* = 3; ^∗∗^*p* < 0.01 and ^∗∗∗^*p* < 0.001). Figure S5: 3D microsphere culture inhibited the apoptosis of MG63 cells in the presence of Dox for 24 hours. (A) The apoptosis of MG63. (B) Relative gene expression of Bcl-2 and Bax in the GelMA microsphere cultured MG63 cells was determined by qRT-PCR (*n* = 3; ^∗^*p* < 0.01 and ^∗∗∗^*p* < 0.001; ns: not significant). Figure S6: different expressed gene analysis of MG63 cell was cultured on MS and TCP and identified pathways potentially regulated by 3D-MS. (A) Heatmap of osteoclast-related and cancer-related genes upregulated by 3D-MS; (B) KEGG pathway analysis of uprelated genes regulated by 3D-MS. Figure S7: 3D microsphere cell culture speeded bone mass loss in the tumor-bearing mice.
