# Supplementary material for: Honeycomb-Like Hydrogel Microspheres for 3D Bulk Construction of Tumor Models
Source: Research (Wash D C). 2022 Feb 7;2022:9809763. doi: 10.34133/2022/9809763 (PMC8848337; doi:10.34133/2022/9809763)
Supplement: Supplementary Materials — Figure S1: the morphological characteristics of the hydrogel microspheres with different GelMA concentrations at 5%, 7.5%, and 10%. (A) SEM observation of the microspheres. (B) Particle size distribution of the microspheres. (C) AFM detection of roughness of the microspheres. (D) Cell counting of osteosarcoma cells cultured in the microspheres for 3 days (n = 3; ∗p < 0.1, ∗∗p < 0.01, and ∗∗∗p < 0.001). Figure S2: fluorescent images for human osteosarcoma MG63 cells cultured in the GelMA microsphere for various periods. (A) The nucleus was stained blue, and the cytoskeleton was stained green (magnification, 50x). (B) Dead cells were stained red, and live cells were stained green (magnification, 50x). Figure S2: fluorescent images for human osteosarcoma MG63 cells cultured in the GelMA microsphere for various periods. (A) The nucleus was stained blue, and the cytoskeleton was stained green (magnification, 50x). (B) Dead cells were stained red, and live cells were stained green (magnification, 50x). Figure S4: K7M2 cells were cultured on MS and TCP for 7 days, respectively. Gene expression of VEGF, Fapα, CXCR3, and MMP-2 in K7M2 cells by qRT-PCR (n = 3; ∗∗p < 0.01 and ∗∗∗p < 0.001). Figure S5: 3D microsphere culture inhibited the apoptosis of MG63 cells in the presence of Dox for 24 hours. (A) The apoptosis of MG63. (B) Relative gene expression of Bcl-2 and Bax in the GelMA microsphere cultured MG63 cells was determined by qRT-PCR (n = 3; ∗p < 0.01 and ∗∗∗p < 0.001; ns: not significant). Figure S6: different expressed gene analysis of MG63 cell was cultured on MS and TCP and identified pathways potentially regulated by 3D-MS. (A) Heatmap of osteoclast-related and cancer-related genes upregulated by 3D-MS; (B) KEGG pathway analysis of uprelated genes regulated by 3D-MS. Figure S7: 3D microsphere cell culture speeded bone mass loss in the tumor-bearing mice. [file 9809763.f1.docx]

**Supplementary Materials**

**Honeycomb-like Hydrogel Microspheres for 3D Bulk Construction of Tumor Models**

Jiachen He^1^^,2*^, Chichi Chen^1*^, Liang Chen^1*^, Ruoyu Cheng^3^, Jie Sun^1^, Xingzhi Liu^4^, Lin Wang^1^, Can Zhu^1^, Sihan Hu^1,5^, Yuan Xue^1,5^, Jian Lu^1^, Huiling Yang^1^ †, Wenguo Cui^3^†, Qin Shi^1,2^†

^1^Department of Orthopedics, the First Affiliated Hospital of Soochow University, Orthopedic Institute of Soochow University, 899 Pinghai Road, Suzhou, Jiangsu 215031, P.R. China.

^2^National Clinical Research Center for Hematologic Diseases, the First Affiliated Hospital of Soochow University, 899 Pinghai Road, Suzhou, Jiangsu 215031, P.R. China.

^3^Department of Orthopaedics, Shanghai Key Laboratory for Prevention and Treatment of Bone and Joint Diseases, Shanghai Institute of Traumatology and Orthopaedics, Ruijin Hospital, Shanghai Jiao Tong University School of Medicine, 197 Ruijin 2nd Road, Shanghai 200025, P. R. China.

^4^School of Nanotech and Nano-Bionics, University of Science and Technology of China, 388 Ruoshui Road, Suzhou, Jiangsu 215123, P. R. China.

^5^Department of Orthopedics, Wuxi Ninth People’s Hospital affiliated to Soochow University, Wuxi, Jiangsu, 214026, P. R. China.

^*^These authors contributed equally to this work

†Corresponding Author(s): suzhouspine@163.com (Huiling Yang), wgcui80@hotmail.com (Wenguo Cui), shiqin@suda.edu.cn (Qin Shi).

**Supplementary Figures 1 to 7**


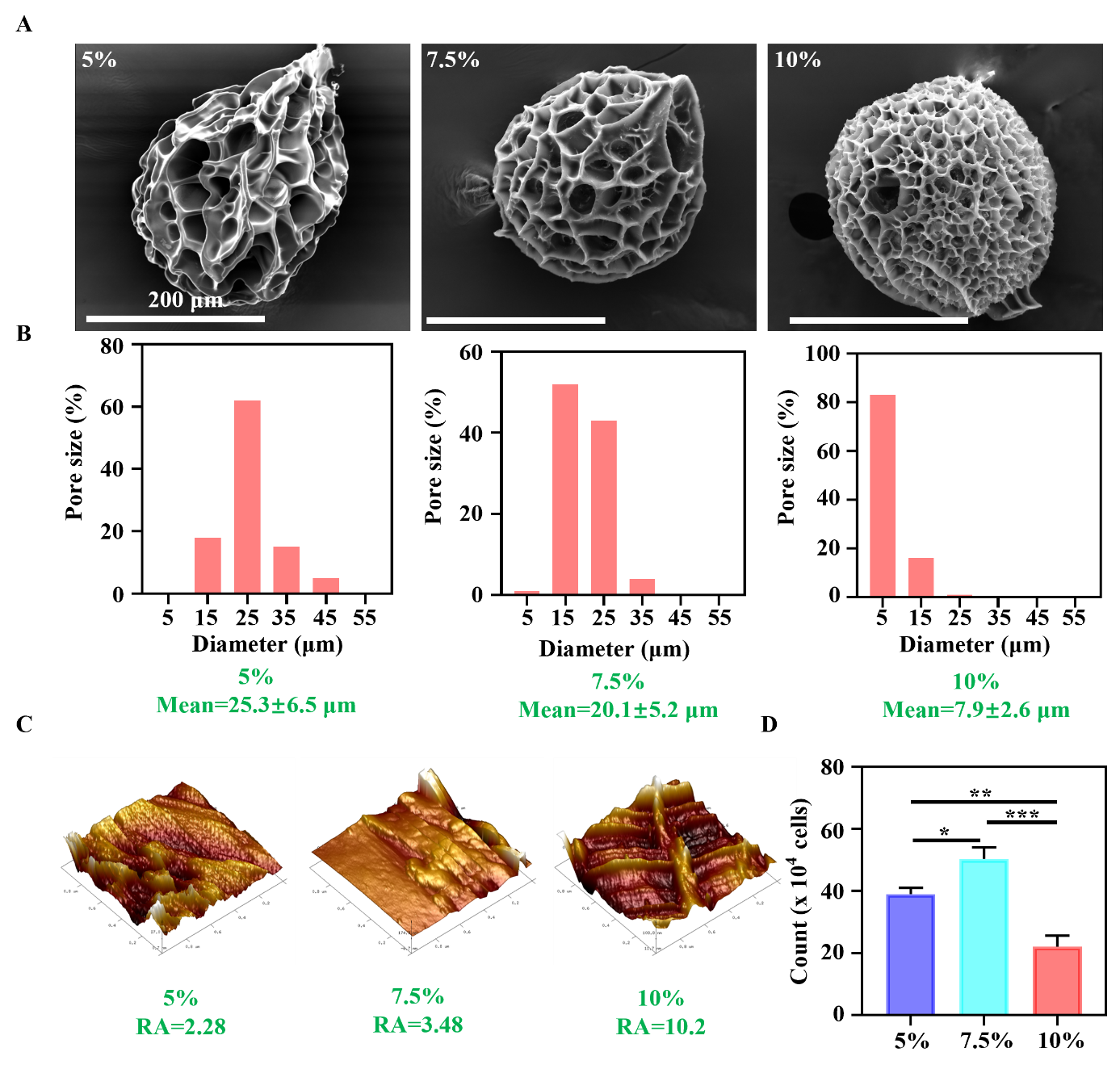


Figure S1. The morphological characteristics of the hydrogel microspheres with different GelMA concentrations at 5%, 7.5% and 10%. (A) SEM observation of the microspheres. (B) Particle size distribution of the microspheres. (C) AFM detection of roughness of the microspheres. (D) Cell counting of osteosarcoma cells cultured in the microspheres for 3 days. (n=3, **p* < 0.1, ***p* < 0.01, ****p* < 0.001).


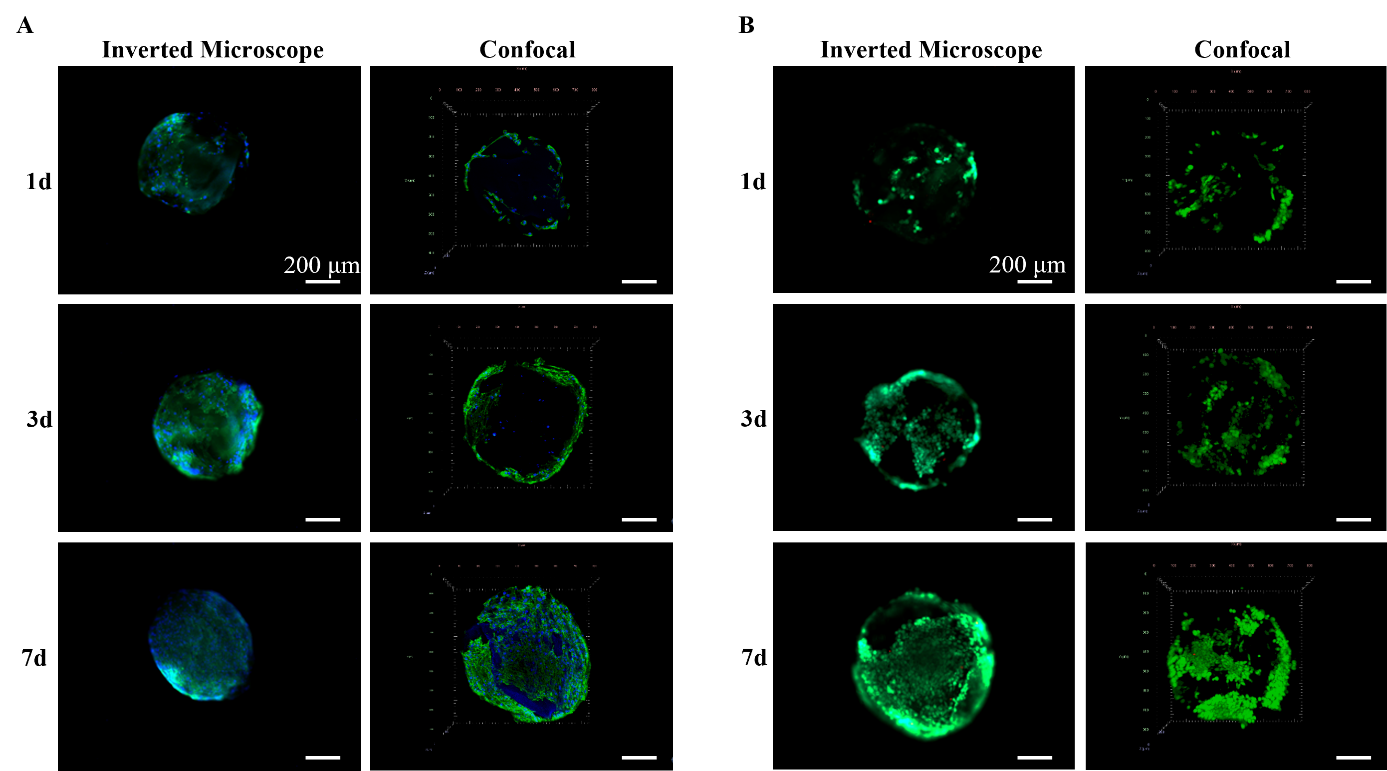


Figure S2. Fluorescent images for human osteosarcoma MG63 cells cultured in the GelMA microsphere for various periods. (A)The nucleus was stained blue, and the cytoskeleton was stained green (magnification, 50×). (B) Dead cells were stained red and live cells were stained green (magnification, 50×).


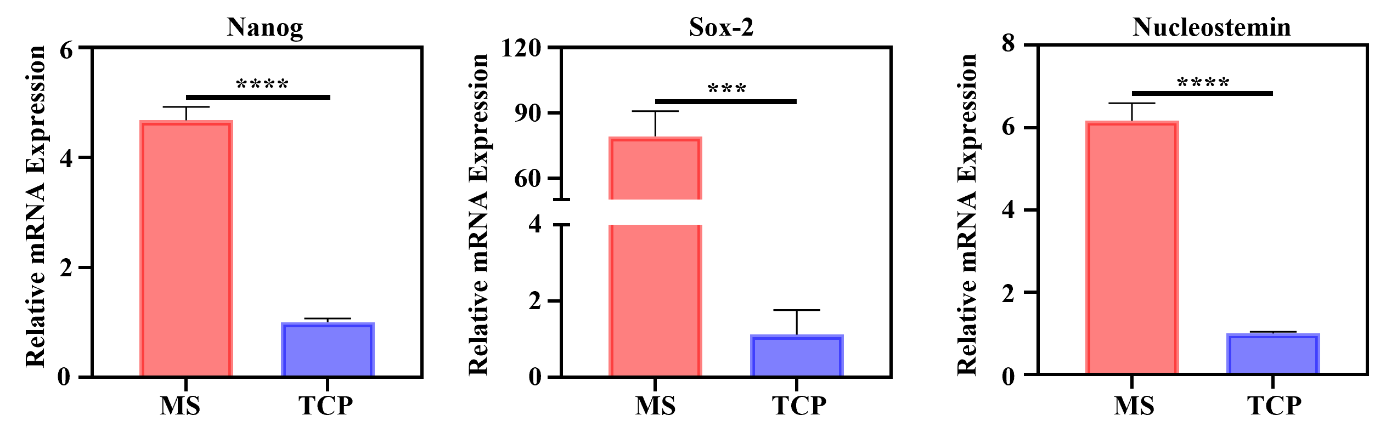


Figure S3. K7M2 cells were cultured on MS and TCP for 7 days, respectively. Gene expression of Nanog, Sox-2, and Nucleostemin of K7M2 cells by qRT-PCR. (*n*=3, ****p* < 0.001, *****p* < 0.0001).


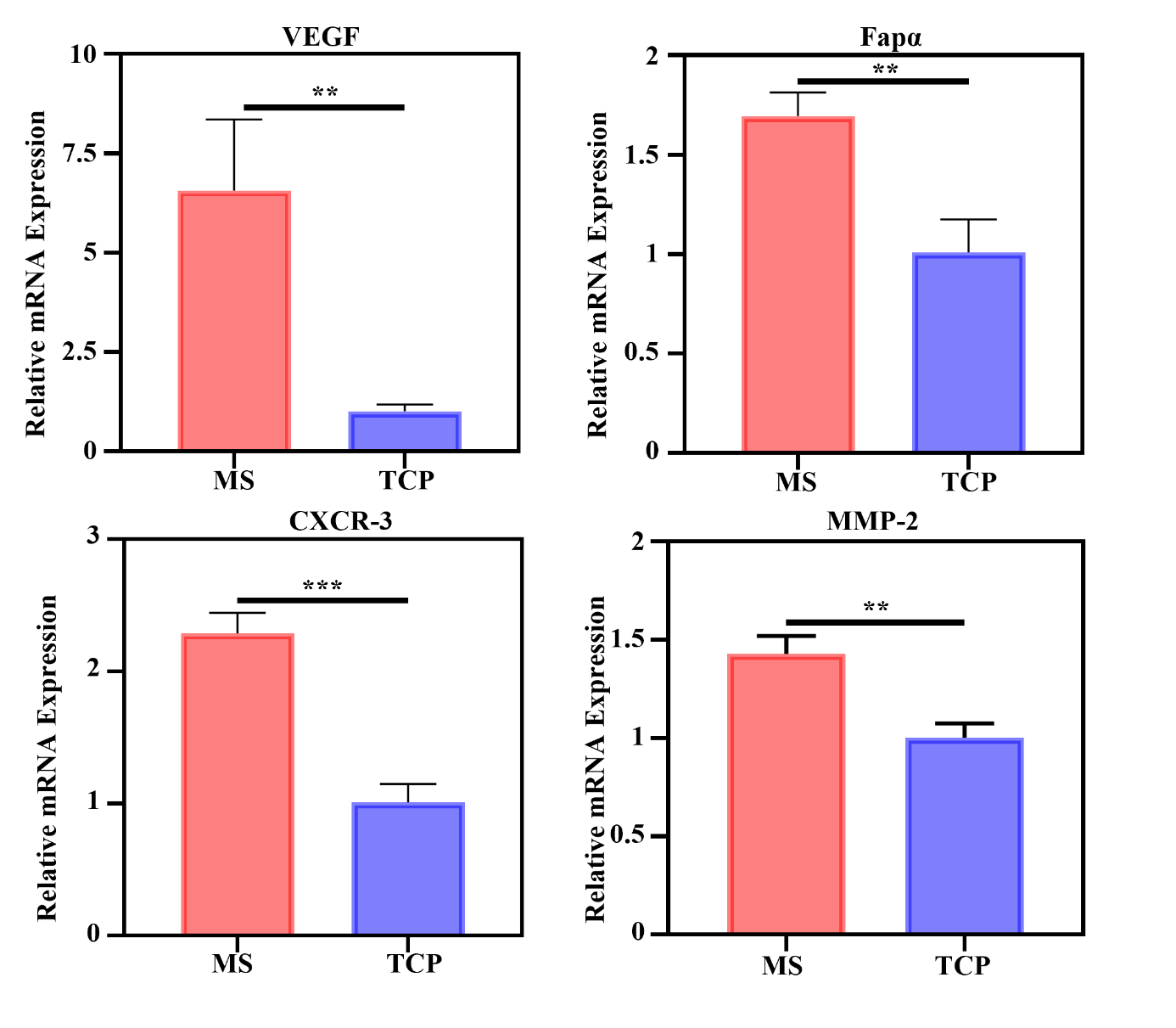


Figure S4. K7M2 cells were cultured on MS and TCP for 7 days, respectively. Gene expression of VEGF, Fapα, CXCR3, MMP-2 in K7M2 cells by qRT-PCR. (*n*=3, ***p* < 0.01, ****p* < 0.001).


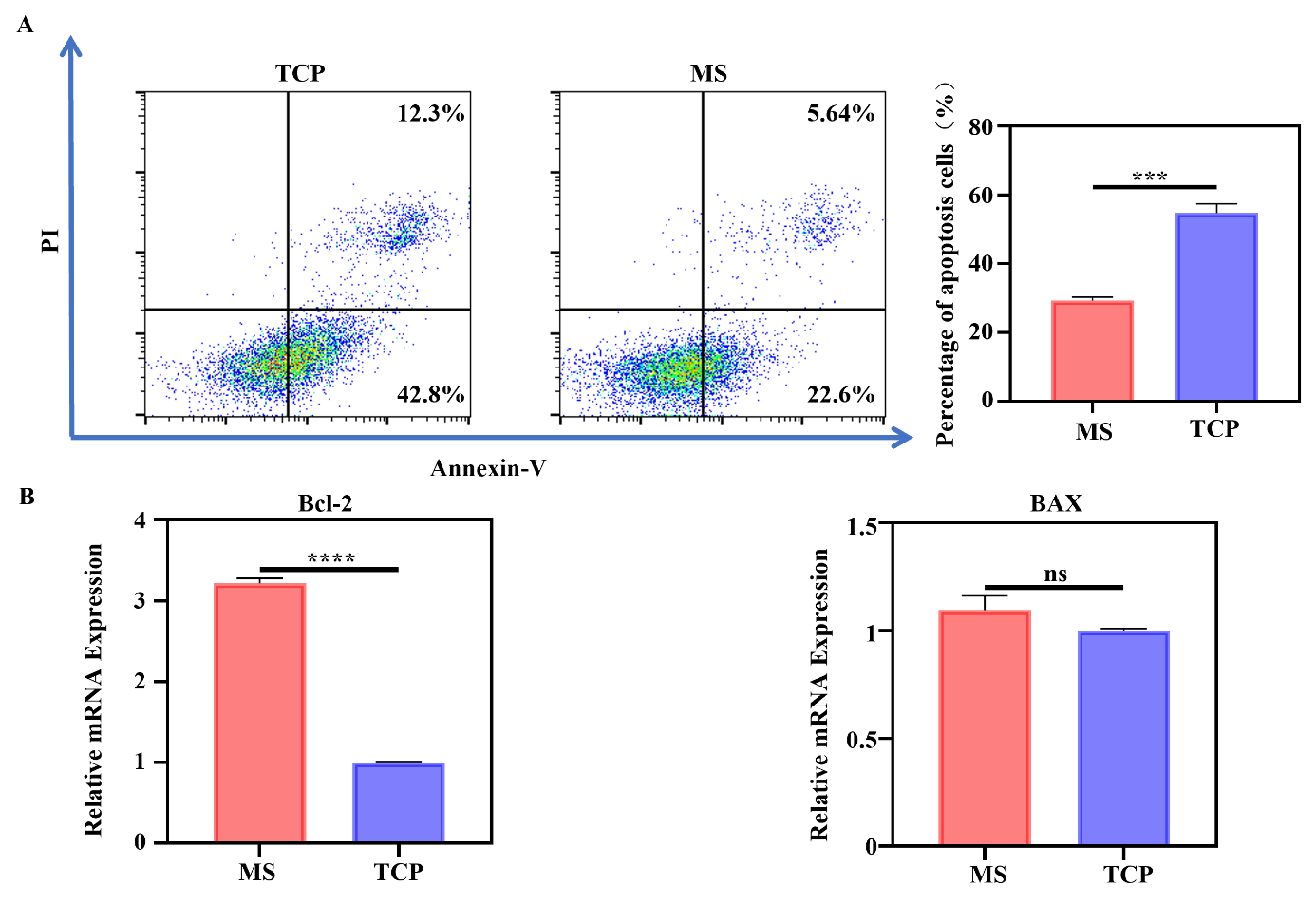


Figure S5. 3D-microsphere culture inhibited the apoptosis of MG63 cells in the presence of Dox for 24 hours. (A) The apoptosis of MG63 (B) Relative gene expression of Bcl-2 and Bax in the GelMA microsphere cultured MG63 cells were determined by qRT-PCR. (*n*=3, **p* < 0.01, ****p* < 0.001, ns, not significant).


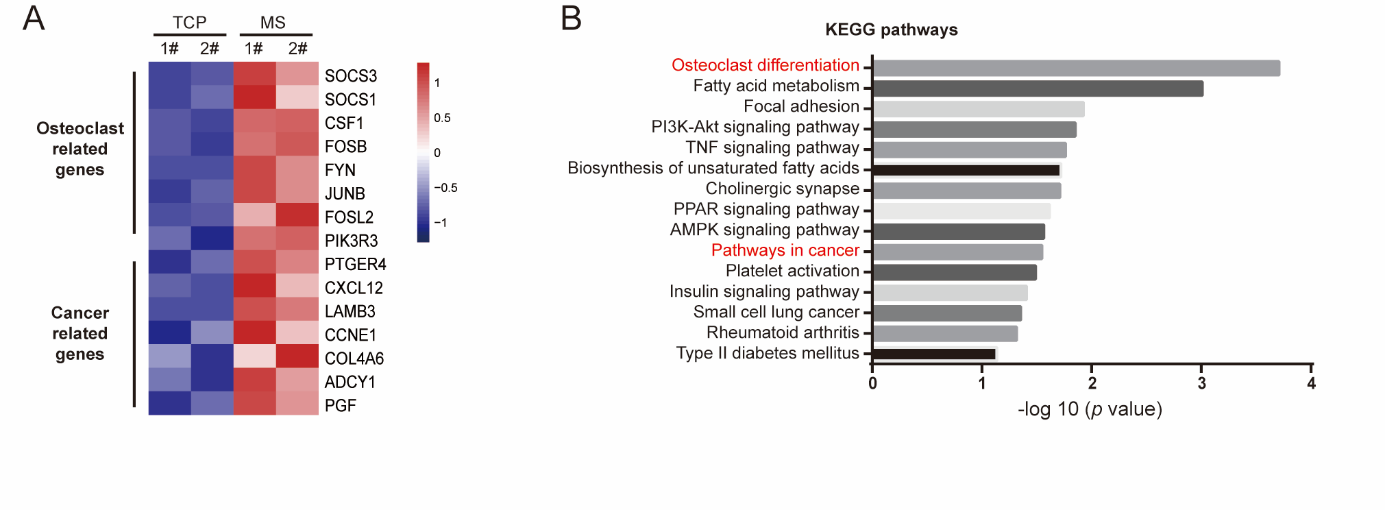


Figure S6. Different expressed gene analysis of MG63 cell was cultured on MS and TCP and identified pathways potentially regulated by 3D-MS. (A). Heatmap of osteoclast-related and cancer-related genes up-regulated by 3D-MS; (B). KEGG pathways analysis of up-related genes regulated by 3D-MS.


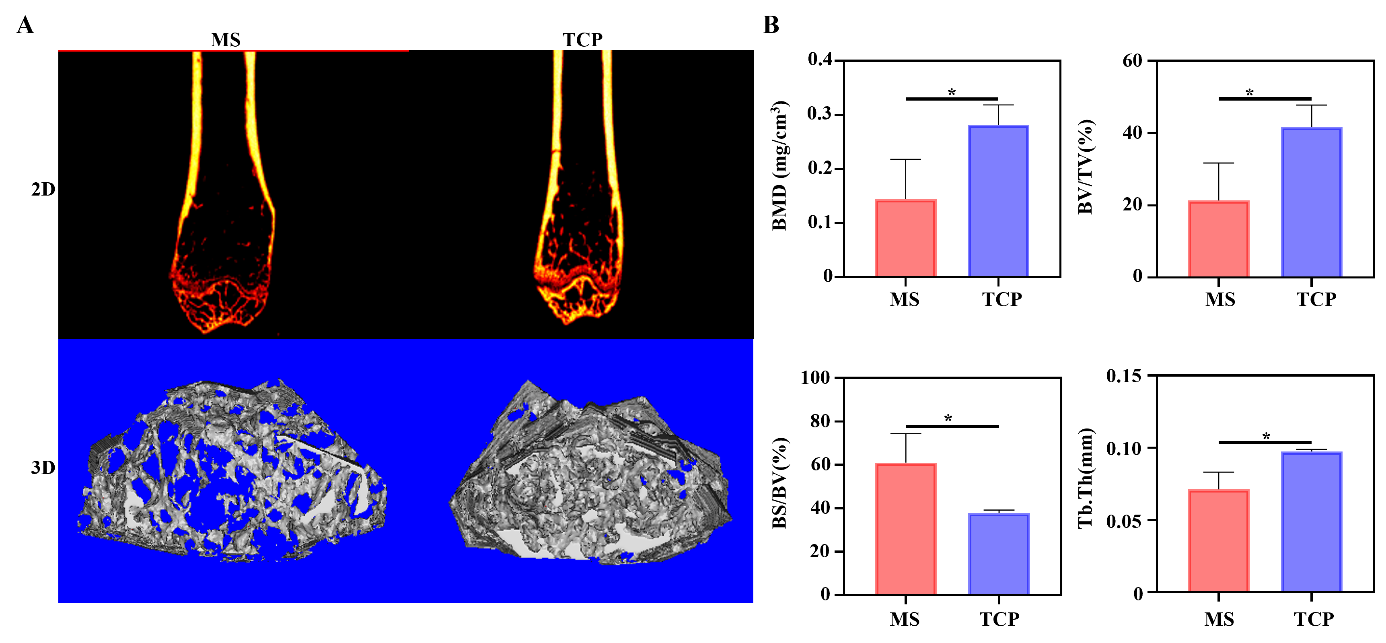


Figure S7. 3D Microsphere cell culture speeded bone mass loss in the tumor-bearing mice. K7M2 cells were cultured on MS and TCP for 7 days and collected, respectively. Mice were implanted with K7M2 cells from MS or TCP culture, respectively. (A) 2D and 3D reconstructions of femurs by Micro-CT scanning. (B) Quantitative analysis of bone parameters of femur Micro-CT analysis, including BMD, BV/TV, BS/BV, and Tb. Th. (*n*=3, **p* < 0.05, ***p* < 0.01, ****p* < 0.001).
